# The interactions between antimicrobial materials and bacterial membranes

**DOI:** 10.3389/fchem.2026.1868096

**Published:** 2026-09-01

**Authors:** Christopher Wilde, Tzong-Hsien Lee, Ketav Kulkarni, Jhih-Hang Jiang, Anton Y. Peleg, Yue Qu, Marie-Isabel Aguilar

**Affiliations:** 1 Department of Biochemistry & Molecular Biology, Monash University, Melbourne, VIC, Australia; 2 Department of Microbiology, Monash University, Melbourne, VIC, Australia; 3 Department of Infectious Diseases, Alfred Care Group and School of Translational Medicine, Monash University, Melbourne, VIC, Australia; 4 Centre to Impact AMR, Monash University, Clayton, VIC, Australia

**Keywords:** antibioflim, antimicrobial materials, antimicrobial pepides, bacterial membranes, conjugated AMPs

## Abstract

Microbial contamination of surfaces in everyday use is an on-going challenge to prevent and eradicate. Furthermore, rapidly growing resistance of microorganisms to many drugs and disinfectants also poses a significant challenge in the design of more effective materials for managing such critical areas as food safety, aged care and agricultural, veterinary and clinical settings in general. Antimicrobial peptides (AMPs) are the primary line of defence of the innate immune system against invading microorganisms and act by binding to and disrupting the outer membranes of microbial cells. Antimicrobial biomaterials also have the potential to transform the current management of microbial contamination. Various polymer-based antimicrobial materials have been developed and many of these are designed to mimic the cationic properties of AMPs or with AMPs grafted onto a polymer template. These antibacterial materials therefore act by membrane disruption. This review provides an overview of the field of antimicrobial materials with a focus on the impact of these materials on bacterial membrane, and aim to provide a timely overview of the molecular mechanism of action of these materials.

## Graphical Abstract

**Figure:**
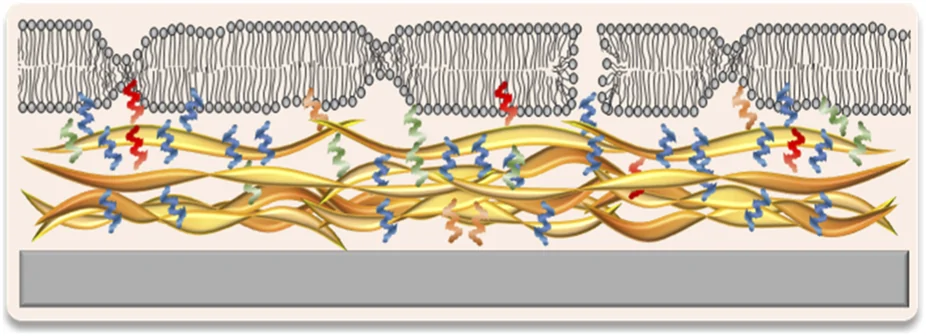
A review of the field of antimicrobial materials with a focus on the impact of these materials on bacterial membranes.

## 1 Introduction

Antimicrobial resistance (AMR) to current antibiotics is increasing at alarming rates (Ahmed et al., 2024; Reza et al., 2025). The control and treatment of high-mortality multi-drug resistance bacterial infection is an urgent issue to be solved for global health and socioeconomics. Due to the bacterial resistance of traditional antibiotics, at least 19 alternative agents/approaches with different mechanism of action were developed and have regularly been assessed in the preclinical and clinical antibacterial development pipeline by the World Health Organization (Czaplewski et al., 2016; Gigante et al., 2024; Rex et al., 2019). Antimicrobial peptides (AMPs) are one class of alternative agents with significant potential for combating AMR, with more than 5,000 examples identified to date (Mookherjee et al., 2020; Wang G. et al., 2026). They exhibit a broad range of antibacterial profiles in terms of potency and selectivity, which provides the opportunity to use both broad-spectrum and narrow-spectrum antibiotics, such as species-specific AMPs in the development of new drugs to eradicate microbial biofilms and treat recurrent infections (Rai et al., 2022).

However, there are significant challenges in the clinical translation of these peptides, ranging from proteolytic degradation *in vivo*, targeting specificity and toxicity to host cells. Despite extensive studies of AMP structure and target selectivity, the mechanism of action (MOA) can vary significantly between AMPs and is poorly characterised for the majority of AMPs identified to date (Lee et al., 2016). Clarification of MOA is essential for the development of new AMP-based drugs. Host toxicity is another important issue and only very few AMPs have been approved for internal use due to host toxicity (Del Olmo and Andreu, 2025; Gomes et al., 2018; Levine, 2006).

The membrane targeting effect of AMPs also holds great potential in the development of novel antimicrobial materials with membrane targeting MOA, with some materials achieving this aim through integration of AMPs within the materials design. Combining multiple AMPs has shown synergistic activity and demonstrated increased potency when combined with conventional antibiotics. While resistance has been shown to develop in response to membrane acting molecules, this combination strategy provides an additional MOA to reduce the risk of AMR development (Hall et al., 2020).

Membrane-targeting strategies are important approaches as they kill bacteria upon contact. This compares to the inhibition of specific cellular protein-, RNA- or DNA-based targets which bacteria are more easily able to overcome, leading to the generation of resistance (Duong et al., 2021; Su et al., 2025). The combination of membrane-targeting strategies with conventional antibiotics delivery also opens up another approach to efficient antimicrobial materials.

Microbial contamination of surfaces continues to challenge the healthcare sector particularly in the area of implantable devices and aged care settings. The targeting of the bacterial membranes by materials has led to many design principles around charge, hydrophobicity, hydrophilicity, and structural design of constituent molecules of materials. In this review, we highlight the advances made in understanding the MOA that materials have with the bacterial membrane. Bacterial membranes can vary greatly in their composition, and the impact of this on the MOA of the materials will be discussed, alongside a discussion of experimental methods of exploring these mechanisms. The review will conclude with a discussion on the challenges of utilising material-based antimicrobials in clinical applications.

## 2 Bacterial membrane structure

**FIGURE 1:**
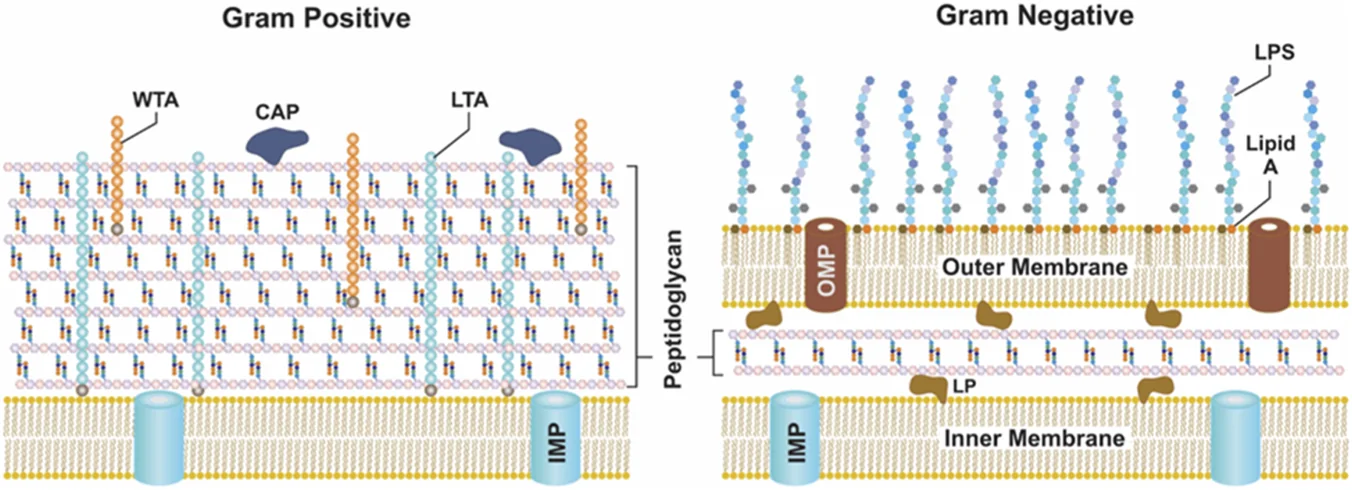
Depiction of Gram-positive and Gram-negative cell envelopes: CAP = covalently attached protein; IMP, integral membrane protein; LP, lipoprotein; LPS, lipopolysaccharide; LTA, lipoteichoic acid; OMP, outer membrane protein; WTA, wall teichoic acid.

Bacteria can be classified into Gram-positive (G+) and Gram-negative (G-) categories based on the structure of the cell envelope (Silhavy et al., 2010). For Gram-positive bacteria, the structures include an outer cell wall composed of peptidoglycan, that surrounds an inner plasma membrane as shown in Figure 1. Gram-negative species have an outer membrane, that encapsulates their peptidoglycan cell wall and surrounds their inner membrane. This arrangement of the membrane strongly influences the interactions that occur between the bacteria and the environment.

The peptidoglycan cell wall provides mechanical support for bacteria, while also being porous allowing the passage of molecules up to 30–57 kDa to diffuse through. The thickness of the cell wall differs between Gram-positive and Gram-negative species, with Gram-positive possessing a thick cell wall around 20–80 nm thick, whereas Gram-negative bacteria have a relatively thin layer less than 10 nm (Mai-Prochnow et al., 2016). Gram-positive cell walls also contain wall teichoic acids (WTA) and lipoteichoic acids (LTA) that impart a net negative charge on the cell wall (Gross et al., 2001). An analysis of bacterial cells has revealed the cell wall is also composed of proportions of amino acids, amino sugars, and sugars connected to peptidoglycan that are distinct between different species (Rohde, 2019). The unique structure of the cell wall is therefore central to evaluating differences in activity of cell-wall-targeting antimicrobial molecules across bacterial species.

The outer membrane (OM) of Gram-negative bacteria surrounds this thin cell wall with proteins anchoring the membrane to the rigid structure (Silhavy et al., 2010). The OM is an asymmetric membrane, with the outer leaflet containing lipopolysaccharide (LPS) and the inner leaflet containing phospholipids (Fivenson et al., 2023). LPS being the core constituent of the outer leaflet is an anionic macromolecule composed of lipid A, a core oligosaccharide and O-antigen repeats. Lipid A has two glucosamine residues linked by a β-1,6 linkage and one phosphate group on each sugar residue and is conserved amongst Gram-negative bacteria. The phosphate groups of lipid A crosslink with the divalent cations Ca^2+^ and Mg^2+^, forming a penetration barrier to hydrophobic compounds. The OM has been shown to be involved in helping Gram-negative bacteria to resist internal turgor pressure (Rojas et al., 2018).

The inner membrane (IM) of Gram-negative bacteria is composed of mainly the zwitterionic phosphatidylethanolamine (PE), making up more than 60% of the membrane lipids, with substantial fractions of phosphatidylcholine (PC) and cardiolipin (CL) (Kralj et al., 2022). The singular plasma membrane of Gram-positive bacteria is made up of majority anionic lipids such as phosphatidylglycerol (PG) and CL, with a smaller proportion of the membrane made up of zwitterionic lipids such as PE (Malanovic and Lohner, 2016).

In addition to lipid composition, lipid bilayers also exhibit topographic features that represent specific regions or domains in which certain lipids and/or proteins are enriched. These domains have been shown to have different susceptibilities to environmental factors including exposure to AMPs (Lee et al., 2021) and are therefore an additional feature that must be considered in defining the structure of a cell membrane.

## 3 Membrane specificity and mechanism of membrane disruption by AMPs

**FIGURE 2:**
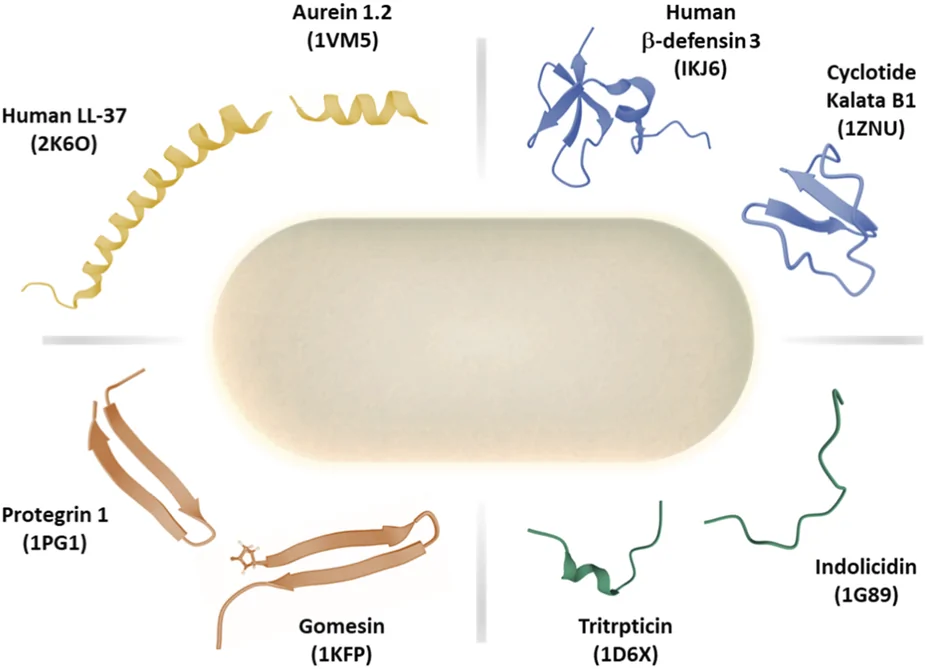
Secondary structures of representative AMPs with the PDB database accession number in brackets.

Many antimicrobial materials aim to mimic the structure and activity of AMPs either through incorporating specific AMPs or designing the material to incorporate similar charge and structure motifs (Haffner and Malmsten, 2018; Li et al., 2023; Rai et al., 2022; Shabani et al., 2024; Yang et al., 2021). AMPs are generally short cationic peptides that are unstructured in solution, and electrostatic forces selectively attract the peptides to the anionic bacterial membrane (Lee et al., 2016). Bacteria also maintain a significant electrochemical gradient that also assists in the targeting of AMPs to bacterial membranes (Lee et al., 2016). Positively charged lysine and arginine residues have been shown to play a role in this attraction, mediating the electrostatic interaction with the negatively charged phosphate moiety of the lipid bilayer (Lee et al., 2019). AMPs also generally undergo a conformational change when they are in close proximity to the membrane, forming a variety of secondary structures (Figure 2) to elicit activity. Helical forming peptides undergo a conformation change from an extended coil conformation, while many β-sheet forming AMPs maintain structural stability in solution and undergo limited structural change upon binding to the membrane (Lee et al., 2019).

**FIGURE 3:**
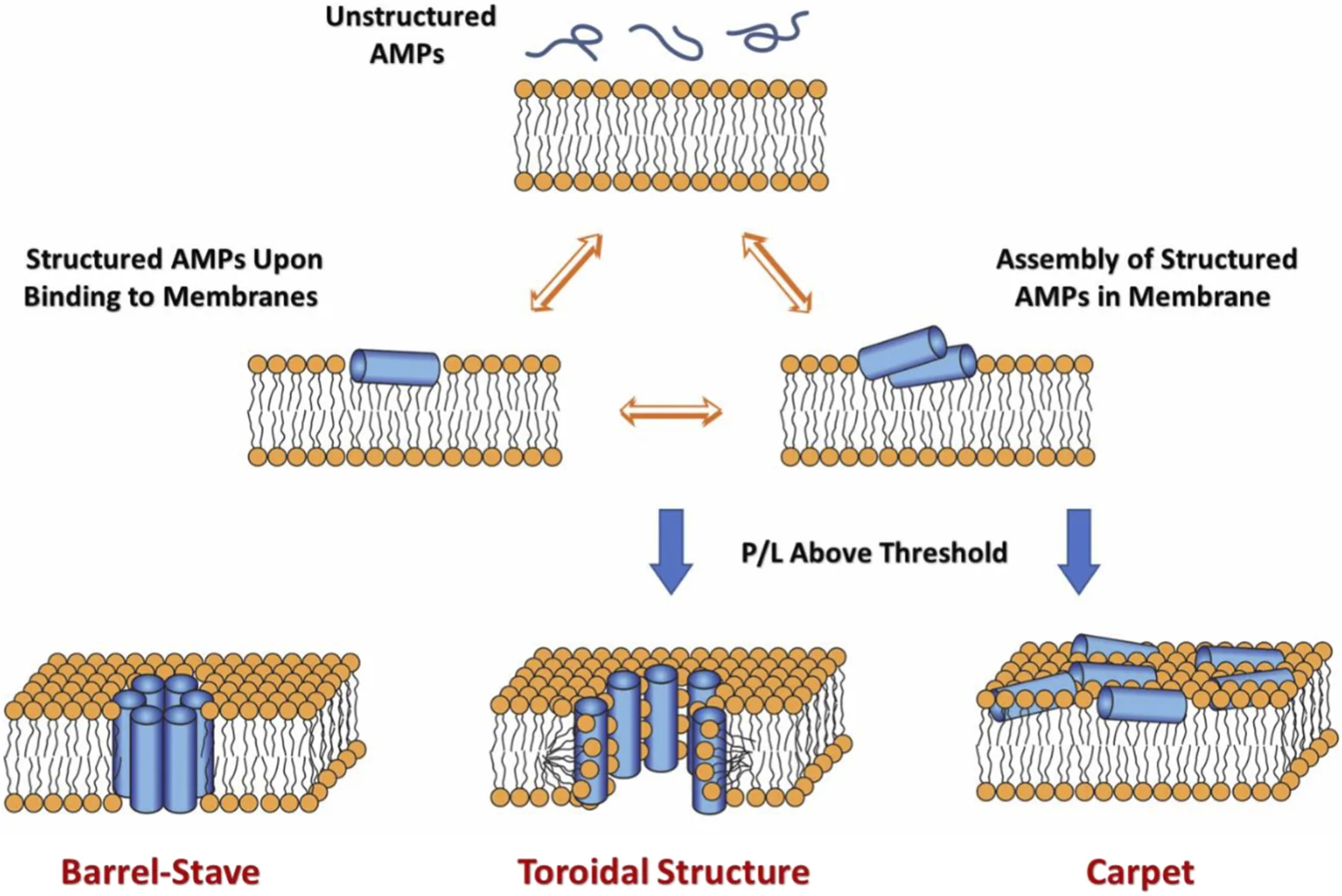
Proposed mechanisms of AMP-mediated membrane disruption including the Barrel-stave pore, toroidal pore and carpet mechanism.

The main mechanisms by which AMPs impact membrane structures can be classified into two categories: membrane disrupting and non-destructive membrane permeability (Melo et al., 2009). The mechanistic actions of AMPs are thought to go beyond just pore formation and to also include the structural reorganization of the membrane (Lee et al., 2016; Lee et al., 2019). The AMP mechanism of action requires a threshold concentration of peptides on the bacterial membrane surface for membrane disruption to cause cell death (Fjell et al., 2011; Mookherjee et al., 2020). Mechanisms of membrane permeabilization have been described by various models as shown schematically in Figure 3. However, the same AMP can exert different mechanistic effects on microbial membranes with varying lipid compositions (Fjell et al., 2011; Lee et al., 2019; Mookherjee et al., 2020). The “barrel-stave” model involves aggregation of AMPs and gradual formation of pores that act as ion channels, destabilising the electrochemical balance of the membrane. The “toroidal pore” model describes the aggregation of AMPs with lipids inducing the bending and stretching of the cell membrane structure due to hydrophobic structures forming between lipids and peptide. A key to this model is the interaction of lipid and AMP leading to formation of transmembrane channels. The “carpet” model proposes the arrangement of AMPs parallel to the membrane structure and gradual re-arrangement of the peptides until a critical threshold is reached. Upon this threshold the overall membrane integrity begins to breakdown, eventually causing the rupture of the cell membrane. The “aggregate” model proposes the dynamic formation of AMP and lipid micelle complexes, creating pores within the membrane (Wimley and Hristova, 2011).

The selectivity of AMPs for bacterial membranes occurs due to the differences in the charge of the host cells. Mammalian cells contain a majority of the neutrally charged zwitterionic lipid head groups, such as phosphatidylcholine (PC) and phosphatidylethanolamine (PE). Mammalian membranes contain much lower or zero negatively charged membrane components such as phosphatidylserine (PS), phosphatidylglycerol (PG), and cardiolipin (CL). This difference in charge, which is responsible for the initial electrostatic interactions of AMPs with the membrane, thus affects the ability of an AMP to disrupt the membrane, and is responsible for the different levels of activity that AMPs show against different species of bacteria that have different lipid membrane compositions (Lee et al., 2016; Mookherjee et al., 2020).

The mechanism of an AMP can also vary depending on the target structure within the bacterial membrane. For example, disruptions to the OM or IM of Gram-negative bacteria could potentially be more sensitive to distinct interactions occurring between an AMP and the membrane (He and Deber, 2024). For Gram-positive bacteria, the AMP must also be able to reach the membrane through the larger peptidoglycan structure of the cell wall or target the cell wall to disrupt the structure of the bacteria (Li et al., 2021). Passive diffusion is the main mechanism by which AMPs pass through the outer peptidoglycan layer to reach their target lipid membrane (Li et al., 2017).

The specificity of AMPs for the bacterial membrane is therefore important in the design of materials that target the membrane, and understanding how the self-assembly of peptides alters the interaction with the membrane is key for producing active and specific antibacterial materials.

## 4 Design of biomaterials for the treatment of bacterial infections

**FIGURE 4:**
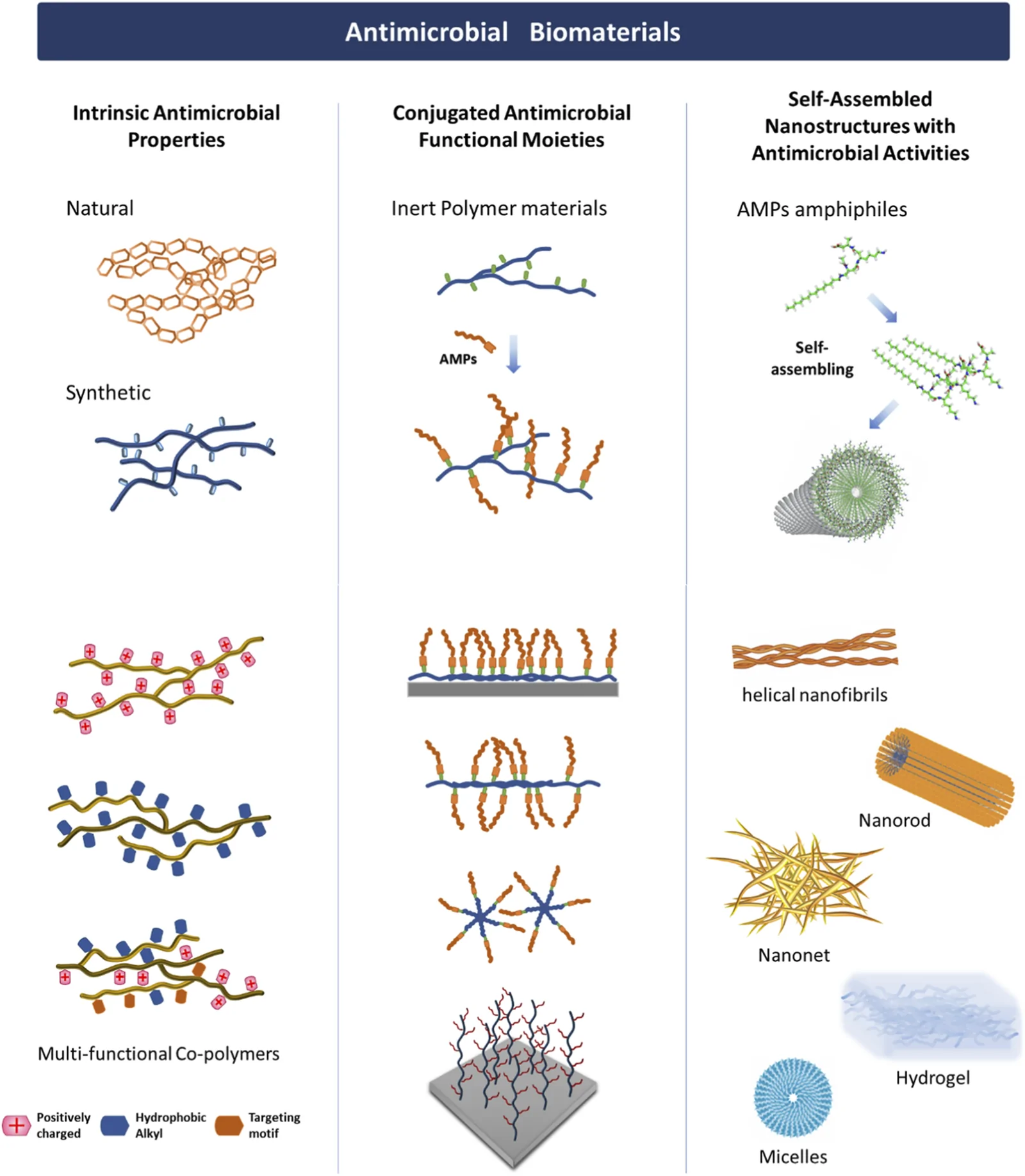
Diagrammatic representation of antimicrobial biomaterials that include polymers with intrinsic antimicrobial activity, inert polymers with antimicrobial moieties and antimicrobial self-assembling materials. Self-assembled peptide materials can adopt various self-assembled architectures.

Biomaterials are engineered to interact with cellular systems to achieve a therapeutic effect and include hydrogels, nanoparticles or microparticles. In the context of antimicrobials, materials can be designed to serve as delivery vehicles for antibacterial drugs/peptides (Martin-Serrano et al., 2019; Wang and Sun, 2021) or be composed of scaffolds and films that have inherent bactericidal or anti-adherence properties (Li et al., 2023; Rai et al., 2022; Salwiczek et al., 2014). Antimicrobial biomaterials are broadly classified according to their biocidal mechanism of action into two principal categories: those that exert their antibacterial effect through the release of biocidal agents into the surrounding environment, and those in which antimicrobial activity is intrinsic to the material’s surface structure, killing microorganisms upon direct contact (Khan and Shakoor, 2023; Nowotnick et al., 2024; Su et al., 2025). In release-based systems, agents such as metal ions, antibiotics, or AMPs are released into the surrounding environment to inhibit bacterial growth. Many of these systems are termed smart materials that respond to environmental stimuli to initiate agent release and have been reviewed in (Monroe and Fikhman, 2023; Wei et al., 2019; Zhang et al., 2023). Intrinsic antimicrobial materials are broadly categorised into three primary classes (see Figure 4): antimicrobial polymers, inert polymers functionalised with antimicrobial moieties, and antimicrobial self-assembling materials (Alkarri et al., 2024; Haktaniyan and Bradley, 2022). This review focusses on the design principles of biomaterials with intrinsic antimicrobial properties that lyse the membranes of bacteria and the reader is referred to a number of excellent reviews that describe different classes of antimicrobial materials (Alkarri et al., 2024; Atefyekta et al., 2021; Haktaniyan and Bradley, 2022; Hall et al., 2020; Li et al., 2023; Lin and Sun, 2022; Monroe and Fikhman, 2023; Muñoz-Bonilla et al., 2019; Rai et al., 2022; Shabani et al., 2024; Su et al., 2025; Takahashi et al., 2023; Wang T. et al., 2026; Yang et al., 2021; Yu et al., 2025a; Zhang et al., 2023; Zou et al., 2020).

### 4.1 Antimicrobial polymers

Polymers possess a great diversity of backbone and chemical structures that can elicit antimicrobial activity. Through modifying hydrophobic and cationic residues within polymers, a great variety of materials with antimicrobial activity can be developed. Antimicrobial polymers can be broadly classified based on their antimicrobial mechanism: 1) antimicrobial polymers with inherent activity within the backbone of the polymer, and 2) inert polymers functionalised with specific antimicrobial groups (Santos et al., 2016). The use of antimicrobial polymers can also vary with polymers capable of being used in solution and covalently attached to surfaces, both of which have an impact on activity (Haktaniyan and Bradley, 2022; Shabani et al., 2024; Si and Chan-Park, 2025).

#### 4.1.1 Antimicrobial polymers with inherent activity

Polymers with inherent antimicrobial activity can be naturally occurring, modified natural polymers or synthetic. Nanostructured antibacterial surfaces have been designed to exploit features at the nanometre scale to prevent bacterial attachment, growth and biofilm formation (Cheng et al., 2019; Tripathy et al., 2017). These materials are inspired by natural templates such as insect wings and plant leaves, and include synthetic arrays of nanopillars or nanotips that rupture the bacterial cell wall via mechanical forces. Moreover, the balance between surface geometry, chemical forces and mechanical rigidity allows control over bacterial adhesion and subsequent biofilm development (Jenkins et al., 2020; Peng et al., 2023). These materials generally demonstrate long-term stability and broad-spectrum efficacy against both Gram-positive and Gram-negative bacteria due to the dominance of physical mechanisms over chemical disruption.

Natural polymers with innate activity such as chitin, chitosan, agarose, carrageen, and *ϵ*-poly-lysine, have long been promising materials with abundance in nature, and modifiable functional groups that can allow for enhancement of activity (Haktaniyan and Bradley, 2022; Muñoz-Bonilla et al., 2019; Takahashi et al., 2023; Teratanatorn et al., 2017). Synthetic antimicrobial polymers generally take inspiration from the amphipathic structure of AMPs, generating polymers with facial amphiphilicity that allows for interactions with and disruption of the bacterial membrane (Lin et al., 2023; Rahman et al., 2018).

Chitosan is a derivative of chitin (β-(1–4)-poly-N-acetyl-D-glucosamine) and is one of the most abundant polysaccharides in nature. Chitosan is produced through the removal of acetyl groups (CH_3_-CO) from chitin. Chitosan is biodegradable, biocompatible, and has low toxicity, that has seen the polymer used in tissue repair and regeneration (Rodríguez-Vázquez et al., 2015). Chitosan also displays broad spectrum antimicrobial and antifungal activity; however, activity is highly dependent on the target microorganism (Ke et al., 2021). The antibacterial activity of chitosan is thought to come from the positive charges of the NH_3_^+^ groups of glucosamine at pH below 6.5 (Raafat et al., 2008). With a greater degree of deacetylation (greater charge) showing increases in antimicrobial activity, primarily driven by increases in electrostatic interactions (Jung et al., 2010). Further modifications to the charge of chitosan can also influence activity with quaternarization of the N atoms of the amino group, primarily through methylation (Martins et al., 2014). Modifying the hydrophobicity of chitosan through substituting a methyl group with a propyl, resulted in an increase in the antimicrobial activity of chitosan (Rúnarsson et al., 2010). Overall chitosan highlights the design principals around natural antimicrobial polymers and enhancing activity through the understanding the effects of charge and hydrophobicity on antibacterial activity.

Hydrogels have also been developed as a wound dressing. For example, a multifunctional composite hydrogel was developed using poly (vinyl alcohol) (PVA)-borax gel as a matrix that was dual-reinforced with dopamine grafted oxidized carboxymethyl cellulose (OCMC-DA) and cellulose nanofibers (Zhong et al., 2021). In addition, an aminoglycoside antibiotic neomycin was incorporated into the hydrogel network as both an antibacterial agent and a cross-linker resulting in a hydrogel with self-healing ability and stretchability and the material was found to be effective against a broad spectrum of bacteria (Zhong et al., 2021).

Synthetic polymers have been developed as antibacterial agents due to their biocompatibility and stability. The synthetic polymers generally contain a balance of cationic and hydrophobic moieties, the balance of which allows for disruption of the negatively charged bacterial membrane (Lin and Sun, 2022; Shabani et al., 2024; Takahashi et al., 2023). The positive charge plays a role in electrostatic attraction with the membrane as seen with the protonated amines of polyethyleneimine (PEI) which are responsible for the bactericidal activity via disruption of the bacterial membrane. The balance of hydrophobic and electrostatic interactions of PEI with phospholipid membranes, leads to the formation of stable defects along model membranes (Sabin et al., 2022). With ultrathin surface coatings of PEI displaying antimicrobial activity against *Staphylococcus aureus* (95% reduction over 24 h) and *Pseudomonas aeruginosa* (80% reduction over 8 h) (Hernandez-Montelongo et al., 2017).

A more recent study showed that the ability of a high molecular weight branched PEI to destabilize the cellular envelope of *E. coli* was significantly reduced following immobilization onto a flat polycarbonate membrane (Andoy et al., 2021). In addition, they showed that PEI immobilized onto a nanoparticle surface interacted differently with bacterial membranes than when immobilized on a flat surface. This study clearly demonstrated how the mode of immobilisation has a strong influence on the interaction between the polymer and the bacterial membrane.”

Optimising the hydrophobicity of synthetic polymers is important for balancing not just antimicrobial activity but also cytotoxicity. Polymers with higher hydrophobicity generally are more toxic to mammalian cells, with no guarantee of an increase in antibacterial activity (Jiang et al., 2022; Kanwal et al., 2025; Phuong et al., 2020). The hydrophobicity of the polymer is still vital to antimicrobial activity and therefore the hydrophobic balance is vital in the design of synthetic polymers. The hydrophobicity can be modulated via the attachment of hydrophobic comonomers such as various length carbon chains (Palermo et al., 2009), indole (Locock et al., 2014), and cholesterol (Coady et al., 2014). Another technique to manage hydrophobicity includes adjusting the lengths of spacers between the cationic and hydrophobic groups of a polymer (Palermo et al., 2012). More recently, AMP-mimicking cationic polymers have been developed showing activity in a mouse model of lung infection (Zhang et al., 2026) and treating multidrug-resistant cancers (Shao et al., 2022). Overall, although modification of the polymer properties can be chemically challenging, modifying the charge and hydrophobicity is vital for the antibacterial activity of natural and synthetic polymers.

#### 4.1.2 Polymers conjugated with antimicrobial moieties

Polymers that are inert can also be functionalised with antimicrobial moieties to develop antimicrobial materials. The AMP CKRWWKWIRW-NH_2_ was immobilized onto a poly (ethylene terephthalate) surface using polyethylene glycol (PEG) hydrogel. The AMP was incorporated into the network using thiol-ene click chemistry in a single polymerization step. The use of D-amino acids improved the metabolic stability of the peptide, with 10 wt% hydrogel displaying bactericidal activity against both *S. aureus* and *Staphylococcus epidermidis* within 1 h (Cleophas et al., 2014).

Atefyekta *et al* immobilized the AMP RRPRPRPRPWWWW-NH_2_ (RRP9W4N) to ordered amphiphilic mesoporous hydrogels made of cross-linked Pluronic F127 triblock copolymer. The covalently bound AMPs did not leach out of the hydrogels and the stability of the AMPs in human serum increased with greater than 50% of the antibacterial activity of the hydrogels retained for 48 h. The hydrogel exhibited broad-spectrum activity against Gram-positive, Gram-negative and even antibiotic-resistant bacteria (Atefyekta et al., 2021).

Hyperbranched polyglycerol (HPG) has been used as an alternative to PEG due to its excellent biocompatibility and HPG conjugates of the AMP aurein 2.2 have been reported (Kumar et al., 2015). Polymers were prepared with varying number of peptides per polymer and circular dichroism confirmed that the aurein 2.2 moiety retained helical structure in the presence of liposomes. The conjugates were active against *S. aureus* and *S. epidermidis* but at a reduced efficacy compared to the unconjugated AMP.

Antimicrobial materials have also been prepared by mixing ureido-pyrimidinone (UPy) based supramolecular polymers with AMPs modified with the same UPy-moiety (Zaccaria et al., 2018). The N-terminus of the AMPs was coupled in solution to an UPy-carboxylic acid synthon resulting in formation of a new amidic bond. Circular dichroism confirmed that the secondary structure of the AMP was retained and the addition of 4 mol% of UPy-AMPs in the UPy-polymer material protected against colonization by *Escherichia coli*, and methicillin-sensitive and -resistant *S*. *aureus* (MSSA and MRSA).

For applications in dentistry an AMP was conjugated with a commonly used monomer for dental adhesive formulation (Xie et al., 2020). Spacer-integrated AMPs were conjugated to methacrylate (MA), and the resulting MA−AMP monomers were copolymerized into dental adhesives as AMP−polymer conjugates. The antimicrobial peptide conjugated to the resin matrix demonstrated significant activity against *Streptococcus mutans*.

Antimicrobial nanoparticles have also been developed in which selenium nanoparticles were coated with the AMP, ε-poly-L-lysine (Se NP-ε-PL) (Huang et al., 2020). Se NP-ε-PL exhibited significantly greater antibacterial activity against eight bacterial species tested, including Gram-positive, Gram-negative, and drug-resistant strains, than their individual components, Se NP and ε-PL. The nanoparticles showed no toxicity toward human dermal fibroblasts at the minimum inhibitory concentrations, demonstrating a therapeutic window.

Cellulose has also been used as a scaffold for the conjugation of AMPs. For example, cellulose-based antimicrobial materials were generated by tethering thioester AMPs and cysteine-modified cellulose and the resulting material was active against *E. coli* (Sperandeo et al., 2020).

Another recent example is the production of neomycin-grafted cellulose-based materials were used as the antibacterial network and a blueberry extract (anthocyanin) as the colorimetric additive to create a dual network gel bandage for smart bandages along with a polyvinyl alcohol/cellulose nanofiber matrix (Yu et al., 2025b).

The same group has also prepared a range of organic–inorganic hybrid materials to create air-permeable and flexible multifunctional cellulose-based textiles with bactericidal activity, thermal heating conversion and electromagnetic interference shielding properties. A conductive MXene was decorated onto polydopamine modified cellulose nonwovens, followed by *in situ* polymerization of glycidyl methacrylate and the resultant PGMA facilitated the covalent grafting of disinfecting agents and also protected the MXene from oxidation and made the materials highly biocidal (Yu et al., 2022).

Overall, for AMPs conjugated to materials, the potential to reduce haemolytic activity, and increase the AMP stability for a longer half-life in serum are potential advantages of AMP-conjugated materials. However, optimisation of peptide tethering process needs to be performed to ensure accessibility of the AMP to interact with the bacteria. This also includes modulation of other important factors such as molecular architecture, amphiphilicity, and spatial arrangement of the tethered AMPs.

### 4.2 Antimicrobial materials based on peptide self-assembly

While a range of polymeric materials are in use, all have several shortcomings in terms of their chemical stability, long-term effectiveness and design flexibility for different surfaces and pathogens (Ikkene et al., 2023; Konai et al., 2018). In contrast, materials produced via self-assembly of peptides allows the bottom-up design of bioactive materials with properties determined by the nature of the monomeric building block (Cross et al., 2021; Levin et al., 2020). The self-assembly of peptides involves the interactions of various non-covalent forces between the self-assembling molecules (Levin et al., 2020; Rai et al., 2022; Wang et al., 2016). These include hydrogen bond (H-bond), hydrophobic interactions, π-π stacking, electrostatic interaction, van der Waals force. Other factors that influence self-assembly include concentration, time, temperature, pH, and ions. H-bonds are essential for peptides to aggregate in solutions and are involved in for formation of typical secondary structures such as α-helix and β-sheet. These interactions generally occur through the amide groups within the backbone of the peptide and carboxyl/amide groups in the side chains.

Hydrophobic interactions drive self-assembly in water. The use of several consecutive hydrophobic residues such as alanine (Ala), Leucine (Leu), Valine (Val), Phenylalanine (Phe) within a sequence in combination with a hydrophilic residue head can therefore result in the formation of various nanostructures. The attachment of alkyl chains to a peptide monomer results in the formation of a peptide amphiphile. The hydrophobic nature of the alkyl chain then drives the self-assembly to generate fibrillar structures (Hendricks et al., 2017; Kulkarni et al., 2019).

π-π Stacking of aromatic rings has also been shown to be a core component in the self-assembly of amyloid formation, with for example the Phe-Phe segment reported to self-assemble into various nanostructures (Hamley, 2023; Smith et al., 2008). Electrostatic interactions between oppositely charged residues have been used to generate secondary structures such as β-sheet, for the formation of larger self-assembled structures. Other interactions include the van der Waals forces that are comparatively weaker compared to other forces, but still playing a role in the stabilisation of self-assembled structures driven by the other forces (Wang et al., 2016).

#### 4.2.1 Nanoarchitectures of self-assembled peptides

The design of peptides has led to the formation of various nanoarchitectures (Figure 4) including nanofibers, nanotubes, nanoribbons, and micelles, that all have the potential in drug delivery, tissue engineering, and use as nanomedicines (Beesley and Woolfson, 2019; Levin et al., 2020; Rai et al., 2022). These materials can exert their activity on cells through inherent activity in the self-assembled structure or through the incorporation of functional modalities. The following sections will cover the formation of peptide self-assembled membrane lytic materials, self-assembled materials that incorporate membrane lytic modalities, and self-assembled AMP delivery systems.

#### 4.2.2 Incorporation of AMPs into self-assembling materials

The incorporation of AMPs into predefined nanostructures has become an important strategy to regulate therapeutic interactions with cells in a controlled and predictable manner. Xu *et al* developed a strategy using a synthetic β-sheet forming peptide of repeating glutamine and leucine subunits called (QL)_6_, that belongs to a class of synthetic multidomain peptides (MDPs). MDPs were designed to mimic AMPs with a global β-sheet structure, with masking of hydrophobic residues upon self-assembly playing a role in reducing cytotoxicity (Xu et al., 2018). Chen *et al* built on this work conjugating melittin (Mel), a highly toxic natural AMP from bee venom to (QL)_6_ forming the peptide (QL)_6_-Mel. (QL)_6_-Mel underwent co-assembly with (QL)_6_-K to form nanofibers that display melittin on the surface in a structurally constrained way. The reduced flexibility of the peptide reduced cytotoxicity while largely persevering activity against *E. coli* compared to free melittin (Chen et al., 2019).

Lombardi *et al* utilised a modified version of the AMP myxinidin (WMR) to generate nanofibers that displayed WMR on the surface. Through the addition of a six-alanine spacer (AAAAAA) and attachment of C19 lipid tail to the C-terminus of WMR they developed the WMR2PA construct. Co-assembly with the peptide amphiphiles 2 PA triggered self-assembly into nanofibers, and the fibres were able to inhibit biofilm formation and eradicate existing biofilms of *P. aeruginosa* (Lombardi et al., 2019).

#### 4.2.3 Self-assembling AMP-Based biomaterials

The main challenges of utilising AMPs as antibacterial agents are cytotoxicity, haemolytic activity, poor stability under physiological conditions and high cost (Luong et al., 2020). Amphiphilicity is a typical feature of AMPs which mediates the interactions with the membrane but can also enable molecular self-assembly via certain secondary structures. The design of self-assembling AMP based biomaterials can therefore exploit a range of structural parameters to control self-assembly and bioactivity.

Lipid chains can be appended to the N-terminus or side chains of cationic peptides to modulate the amphiphilicity, generating lipopeptide-like antibacterial agents. The cationic amino acid side chains bind to the membrane, while the hydrophobic lipid tail mediates insertion into and destabilization of the lipid bilayer. Cationic peptide amphiphiles (PAs) have been designed to incorporate AMP inspired sequences with alkyl chains of various lengths (C12-C20) for use as antimicrobial agents (Rodrigues de Almeida et al., 2019; Rounds and Straus, 2020). The PAs undergo self-assembly to form micelles, nanofibers and nanoribbons. The micelle nanostructure was found to be the most potent, with the hypothesis being that micelles are a less stable structure, that can allow the micelle PAs to disassemble to insert into the membrane to cause lysis. Longer alkyl chain lengths were determined to generally increase the activity of the PAs (Rodrigues de Almeida et al., 2019). Peptide amphiphiles have also been designed to self-assemble into antimicrobial peptide hydrogels to treat *Helicobacter pylori* infections (Gong et al., 2024) and as anti-biofilm agents against *A baumannii, P. aeruginosa* and *S aureus* (Cai et al., 2025).

Liu *et al* reported self-assembling peptides made from the 2 AMP units of (KLGAKI)_3_-NH_2_ linked by a tetra peptide linker. The peptide can undergo reversible transformation from a random coil structure to β-folded structure and undergo further self-assembly to form a hydrogel. The self-assembled structure produced nanofibers with AMP sequences located on the external surface of the fibre (Liu et al., 2013).

Xie *et al* explored the effect of chirality on the activity of the amphiphilic AMP (C16-V_4_R_4_), generating two homochiral AMPs (all L- or all D-amino acids) and one heterochiral AMP with alternating D-/L- amino acids, C16-^D^V_4_^L^R_4_. The peptides self-assembled to form nanofibers with the study finding that the chirality of the VVVV portion of the AMP directed the handedness of the nanofiber, with the heterochiral peptide demonstrating a higher degree of aggregation into nanofibers with a lower degree of twist. The heterochiral nanofibers outperformed both the homochiral peptides against both Gram-positive and Gram-negative bacteria. The superior activity of the heterochiral peptide was attributed to its stronger membrane disrupting ability (Xie et al., 2022).

The dipeptide diphenylalanine (Phe-Phe) self-assembles to form nanotubes with potent broad-spectrum activity. Unlike AMPs that are generally longer and contain cationic residues, the short, neutral and aromatic residues can exert antibacterial activity. The dipeptide permeates the outer membrane and leads to depolarization of the inner membrane, mixing Phe-Phe with agar and gelatin to generate a composite hydrogel demonstrating inhibition of bacterial cell growth (Schnaider et al., 2017). Dipeptides have also been used to generate antibacterial nanofibers through metal-mediated crosslinking (Ghosh et al., 2024).

Veiga *et al* developed an arginine-rich self-assembling β-hairpin peptide that forms polycationic fibrillar network. The resulting hydrogels displayed strong antibacterial activity against both Gram-positive and Gram-negative bacteria, with the activity occurring through a membrane disrupting affect. The initial peptide demonstrated haemolytic activity, however through reducing the arginine residue, the haemolytic activity was reduced and only a slight decrease in antibacterial activity was observed (Veiga et al., 2012).

A class of self-assembling β3-tripeptide amphiphiles have been engineered around the formation of 14-helix formation of β-peptide monomers in aqueous solution (Del Borgo et al., 2013; Habila et al., 2020; Williams-Noonan et al., 2024). The addition of N-terminal acylation triggers head-to-tail (1D) self-assembly into fibres via a 3-point hydrogen bonding motif (Kulkarni et al., 2016; Kulkarni et al., 2019). The length of alkyl chain and positional attachment to the β-peptide monomer plays an important role in determining the resulting nanostructure. Attachment of either C14 or C16 to the first residue promotes the formation of twisted fibril structures and changing of the alkyl chain position to the second residue results in the formation of nanobelts (Kulkarni et al., 2016; Kulkarni et al., 2019). In aqueous media the N-acetylated lipidated β3-Tripeptides rapidly self-assemble into fibres and form hydrogels. (Kulkarni et al., 2019).

**FIGURE 5:**
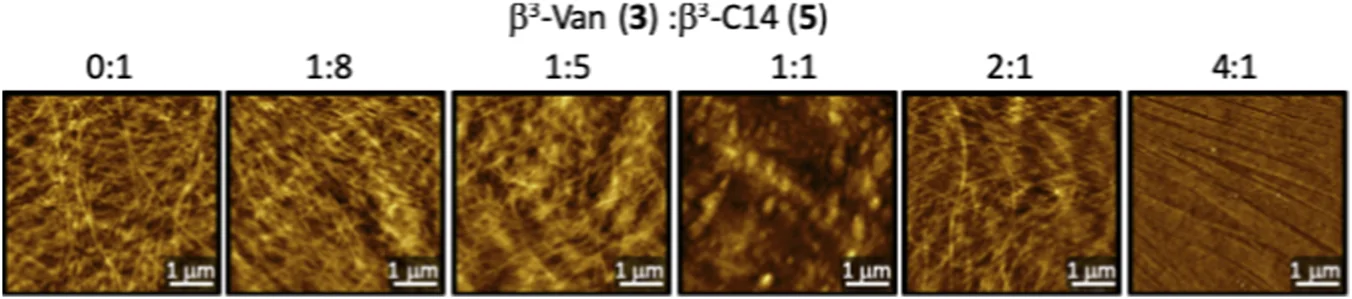
AFM of the nanofibers composed of mixing the two peptides β3-Van:β3-C14 in different ratios. (adapted from (Payne et al., 2021)).

N-acetylated lipidated β3-Tripeptide monomers can be decorated with biological functionality through the incorporation of a unique amino acid with an orthogonal protecting group (Kulkarni et al., 2016). The addition of functional groups likely has limits to the size and hydrophobicity of groups that can be attached without impacting the self-assembly. Co-mixing of functionalised and unfunctionalized β3-tripeptide monomers can be mixed to offset the impact of larger and more hydrophobic groups on the self-assembly mechanism. The conjugation of the antibiotic vancomycin onto a β3-tripeptide monomer has been explored (Payne et al., 2021). While it was still possible to conjugate vancomycin to the N-acetylated lipidated β3-tripeptide monomer, the resulting peptide was very hydrophobic and difficult to dissolve. Therefore, vancomycin was conjugated to the β-peptide monomer in the place of the C14 chain to generate a new β-peptide, β3-Van. Co-mixing with the unfunctionalized peptide (β3-C14) in ratios of β3-Van:β3-C14 of 4:1 demonstrated the formation of thin fibres ∼0.75 nm, suggesting the formation of a singular nanorod (Figure 5). Increasing the ratio of β3-C14 led to the formation of larger and more bundled fibres, indicating less inhibited self-assembly due to the presence of vancomycin. Finally, the antimicrobial activity of the fibrous mesh inhibited the growth of *S. aureus* (Payne et al., 2021). More recently, biomimetic peptide nanonets have been developed based on the self-assembly of the RFQF_4_ derived from human defensin (Gao et al., 2024).

#### 4.2.4 Self-assembling peptides as delivery vehicles

Combining AMPs with delivery platforms can selectively deliver AMPs to the site of infection, protecting AMPs from degradative enzymes, and controlling the release of AMP at the target site. LL-37 is a human cathelicidin peptide that has been considered as an alternative to antibiotics and plays a role in human immune defence (Najmi et al., 2020). Rashki *et al* fabricated Chitosan-nanoparticles (CS-NPs) through crosslinking with sodium tripolyphosphate (TPP) in the presence of LL-37 to generate CS/LL-37-NPs. The encapsulation efficiency of LL-37 in this delivery system was 86.9%, with incorporation of LL-37 within the delivery system prolonging the half-life of the peptide, with maintained activity over 72 h compared to free LL-37 that demonstrated loss of activity after 24 h. CS/LL-37-NPs achieved 68% biofilm inhibition compared with ∼20-fold reduction seen for LL-37 and CS-NPs alone. This reduction was achieved through a significant reduction of icaA gene expression on the surface of CS/LL-37-NPs [103].

De Soricellis *et al* developed a stable nanoparticle formulation of LL-37 load poly (lactide-co-glycolide) PLGA nanoparticles NPs for enhancing wound healing. The N-terminus of LL-37 was modified through the addition of a palmitoyl chain, that was designed to improve the solubility of the peptide, facilitate encapsulation into the PLGA nanoparticles, and enhance its interaction with biological membranes. Using a microfluidic technique to produce the nanoparticles led to superior control over the nanoparticle size and uniformity, that resulted in formulations with reduced cytotoxicity (De Soricellis et al., 2025).

Another strategy with wound healing applications is the use of the peptide L5 which readily forms a hydrogel (Liu et al., 2024). L5 exerted a pH-switchable antimicrobial effect (pH 5.5) and formed biocompatible hydrogels at neutral pH (pH 7.4) with the antimicrobial agent LysSYL. The L5@LysSYL hydrogels increased thermal stability, and exhibited the slow-release effect of LysSYL. Effective elimination of *S. aureus* was observed and promoted wound healing in a mouse model of wound infections caused by MRSA.

D’ Angelo *et al* also prepared PLGA-NPs containing colistin as a model cationic AMP and then surface-modified with either chitosan (CS) or poly (vinyl alcohol) (PVA) to optimise nanoparticle interactions with mucus or biofilms. The resulting nanoparticle achieved ∼63% encapsulation efficiency and sustained release for 15 days. With both CS and PVA variations of NP penetrating *P. aeruginosa* biofilm and maintaining anti-biofilm activity for 72 h, whereas free colistin lost activity after 24 h (D’Angelo et al., 2015).

The non-natural cationic AMP SET-M33 confers a branched structure that provides resistance to degradation in biological fluids and has been incorporated into a nano system (M33-NS) through loading on single-chain dextran nanoparticles (DXT-NPs). M33-NS showed relatively low cytotoxicity against animal cell lines and retained antimicrobial activity against *P. aeruginosa*. The incorporation into the nano system greatly prolonged the residence time of AMPs in the lungs and showed higher local concentrations in pulmonary administration (Falciani et al., 2020).

In summary these studies demonstrate the ability of AMPs to self-assemble into nanocarrier systems for the delivery of AMPs to the site of infection. In particular, the incorporation into nanoparticles greatly increased the retention of AMPs through modulating the release from the nanoparticles.

## 5 Biophysical studies of the interactions between antimicrobial materials and the bacterial membrane

The activity of each material depends critically on the accessibility of the active part of the AMP that can be compromised by attachment to a scaffold or the self-assembly process. These geometrical constraints will then impact on the ability of the AMP to bind to and penetrate the membrane thereby reducing the apparent activity of the material. However, the local concentration of the AMP can be higher as the immobilisation process concentrates the AMP at the target site.

In spite of the importance of defining the mechanism of interaction between the antimicrobial scaffold and the bacterial membrane, there are very few biophysical studies that have focussed on the mechanism in terms of a detailed analysis of the changes in membrane structure. When a material demonstrates antibacterial activity, it is assumed that the AMP positive activity is due to the inherent activity of the AMP or the AMP mimic. However, the pathway from AMP binding to membrane disruption and destruction may change depending on the composition of the bacterial membrane and the environmental conditions. In addition, without a defined mechanism, it is not possible to rationalise a negative result and progress the material design process towards an effective material.

A broad range of biophysical techniques have been used to characterise the effect of AMPs on membrane structure and have been reviewed recently (Lee et al., 2026). These techniques include different spectroscopic techniques, imaging, calorimetry, light scattering, optical biosensors and computational methods. A wide range of spectroscopic and imaging techniques have also been used to analyse biomaterials (Prasad et al., 2024). However, in the context of structural analysis, biomaterials are more commonly imaged using scanning electron microscopy or atomic force microscopy (AFM). However, it is challenging to obtain high resolution structural information of either biomembranes or biomaterials, and extremely difficult to analyse the complex interaction between a biomaterial and a bacterial cell at atomic level.

The interaction of a wide range of polycationic nanoparticles with model membranes was studied a number of years ago using AFM to image the changes in membrane morphology upon exposure to the nanoparticles (Hong et al., 2004; Hong et al., 2006; Leroueil et al., 2007; Leroueil et al., 2008). The results demonstrated that the nanoparticles induced the formation of holes, membrane thinning and in some cases bilayer disruption. These studies were also correlated with dye diffusion and enzyme leakage studies on live bacterial cells. While these nanoparticles were not designed as antimicrobial materials but rather for delivery applications, these studies demonstrated the importance of monitoring the impact on membrane structure and function in order to fully define the mechanism of membrane interaction.

More recently, an antimicrobial methacrylate copolymer was shown to induce domain formation in model membranes (Yasuhara et al., 2022). Using differential scanning calorimetry and fluorescence microscopy, the separation of domains was clearly observed in giant unilamellar vesicles (GUVs) comprised of POPE and POPG. The impact of the incorporation of either methyl methacrylate (MMA) or butyl methacrylate (BMA) into the copolymer provides further insight into the mechanism of membrane disruption. In particular, it was observed that the polymer with BMA formed pores in the lipid bilayer while the copolymers with MMA caused the GUVs to burst, demonstrating a clear role for the alkyl chain moiety in antimicrobial activity (Tsukamoto et al., 2021).

**FIGURE 6:**
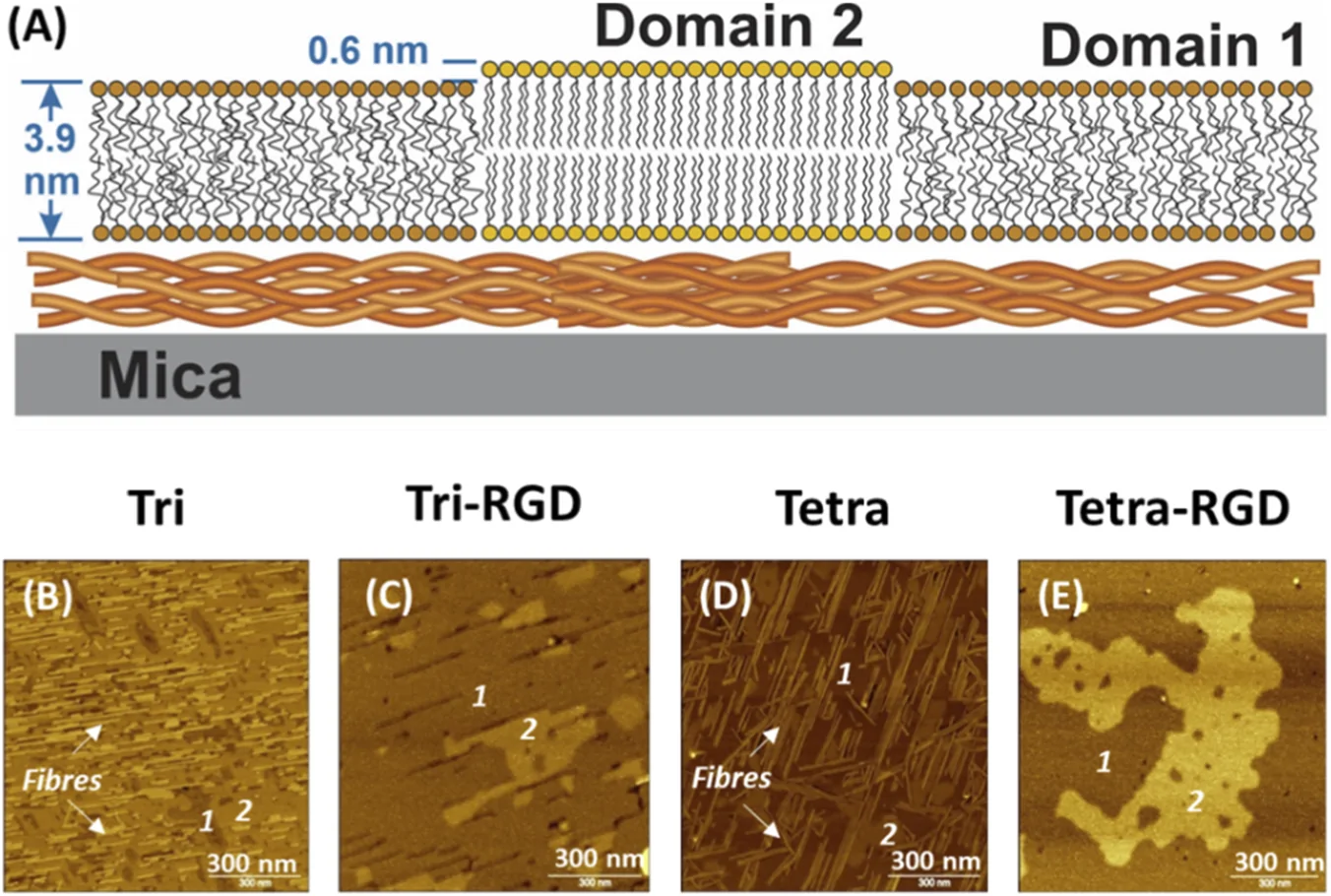
Topographical analysis of POPC/POPE/POPS (6:3:1) supported lipid bilayers on four different β-peptide fibres. **(A)** Schematic depicting the design of the supported lipid bilayer on β-peptide fibres with membrane domains of different thickness and lipid order, **(B)** Tri-β-peptide **(C)** Tri-RGD-β-peptide, **(D)** Tetra-β-peptide **(E)** Tetra-RGD-β-peptide. Fibrils are prominent in **(B,D)** while lipid domains 1 and 2 are present in all four systems. Reproduced with permission from (Wilde et al., 2025).

High resolution imaging of membrane structure changes can be performed by AFM as demonstrated recently for the deposition of model membranes on β-peptide-based fibers containing the RGD cell adhesion motif (Wilde et al., 2025). As shown in Figure 6, two distinct domains of different height were clearly evident and the study demonstrated that the presence of the RGD motif enhanced the stability of the higher domain 2. This system will be well suited to investigate the impact of antimicrobial materials on bacterial cell walls.

In addition to molecular interactions, bacteria are also mechanically resistant and can sustain physical stress. However, high aspect-ratio nanopillars have been shown to deform bacterial cells upon contact and if the deformation is sufficiently large, cell wall lysis occurs and ultimately leads to cell death. Antibacterial surfaces have thus been fabricated to exploit this phenomenon referred to as mechano-bactericide technology. In order to identify design criteria, a recent study demonstrated through modelling that bacterial killing caused by external forces on nanopillars is influenced by surface topography and cell biomechanical variables, including the density and arrangement of nanopillars, in addition to the cell wall thickness and elastic modulus (Valiei et al., 2020; Valiei et al., 2024). Experimentally, it was shown that nanopillars with a larger spacing increase bacterial susceptibility to mechanical puncture (Valiei et al., 2024). More recently, using silicon nanowires, it has been shown that the key parameters for bacterial killing are the sharpness and the pitch size (spacing) (George et al., 2025).

## 6 Conclusions

There is a wide range of antimicrobial materials designed to mimic the action of membrane-lytic AMPs. The design strategies include 1) polymers that mimic the positive charge of an AMP, 2) the conjugation of an AMP to a scaffold, 3) hydrogels and nanoparticles formed via the self-assembly of AMPs and 4) hydrogel-encapsulated AMPs. The applications of these materials include their use as antimicrobial and/or anti-biofilm agents and can be used as coatings or administered as antimicrobial drugs.

There are many challenges to be met particularly in light of the constantly emerging phenomenon of AMR which requires new approaches to biomaterial design. These challenges include the design of a stable material which is effective against a range of bacteria yet non-toxic to mammalian hosts, and the ability to define the mechanism of membrane rupture for each target microorganism. Moreover, depending on the structural template, important factors such as molecular architecture, amphiphilicity and spatial arrangement of the AMP moieties must be modulated to ensure accessibility of the cytolytic agent. Antimicrobial materials also need to be responsive, safe and flexible in design to be applied to a range of physical settings, with minimal risk of driving the emergence of AMR. Recent examples of modular design strategies include multivalent coiled coil peptides (Thota et al., 2021) and bottom-up mix-and-match self-assembled peptides as antimicrobial materials.

It is now evident that the interaction of an AMP can differ depending on the physical properties of the bacterial membrane, and it is not yet possible to establish a definitive structure-function relationship for the interaction between the biomaterial and the bacterial membrane. However, lipidomics using high resolution mass spectrometry now allows the detailed lipid composition of a membrane extract to be determined and given the complexities of bacterial membrane composition and properties, tailoring materials for specific bacteria in specific settings will significantly expand the material armoury to control infections.

While microbial infections within a host are still inaccessible to conventional biophysical analysis, imaging technologies, such as AFM, that are compatible with solution-based bacterial samples, continue to evolve in terms of high-resolution capability (Borrelli et al., 2025). In addition, the emergence of more powerful computational methodologies, from simulations to artificial intelligence, will allow more predictive capacity in antimicrobial material design as demonstrated recently (Jiang et al., 2025).

Integrated experimental and computational strategies are therefore required to address the following questions:

1. Do all AMP-conjugated and AMP-mimic materials interact with bacteria in the same way?
2. How do the geometrical constraints of AMP attachment to a scaffold influence the material interaction with the bacteria?
3. How does the nanoarchitecture of the material influence antimicrobial activity?
4. Does the interaction of a material vary with different bacteria due to the differences in bacterial membrane composition?

The answers to these question will then allow the following challenges to be addressed:

1. What are the key structural parameters regulating the relationship between material structure and antibacterial activity?
2. Are the modes of action derived from model systems relevant to *in situ* mechanisms?
3. In order to minimise the evolution of AMR, can we exploit different lytic mechanisms in material design to prime a lipid membrane with one AMP to enhance susceptibilty to a second antimicrobial compound, as recently demonstrated in an antifungal application (Duong et al., 2021; Simm et al., 2024)?

Finally, in addition to the design and analysis challenges described above, the translation of novel antimicrobial materials into clinical applications requires careful consideration of host-associated infection microenvironments, local and systemic toxicities, and commercial and regulatory imperatives, all of which must be addressed with respect to material stability, durability, safety, manufacturability, regulatory compliance, and consumer acceptance. (Hall et al., 2020; Li et al., 2023; Yang et al., 2021).

In summary, new innovative design platforms must be developed for antimicrobial materials which are multifunctional, have long term chemical and mechanical stability and can be engineered to target different bacteria through a molecular understanding of the material-membrane interaction.
